# Assessing Social Networks in Patients with Psychotic Disorders: A Systematic Review of Instruments

**DOI:** 10.1371/journal.pone.0145250

**Published:** 2015-12-28

**Authors:** Joyce Siette, Claudia Gulea, Stefan Priebe

**Affiliations:** Unit for Social and Community Psychiatry (WHO Collaborating Centre for Mental Health Service Development), Queen Mary University of London, Newham Centre for Mental Health, London, E3 8SP, United Kingdom; Nagoya University Graduate School of Medicine, JAPAN

## Abstract

**Background:**

Evidence suggests that social networks of patients with psychotic disorders influence symptoms, quality of life and treatment outcomes. It is therefore important to assess social networks for which appropriate and preferably established instruments should be used.

**Aims:**

To identify instruments assessing social networks in studies of patients with psychotic disorders and explore their properties.

**Method:**

A systematic search of electronic databases was conducted to identify studies that used a measure of social networks in patients with psychotic disorders.

**Results:**

Eight instruments were identified, all of which had been developed before 1991. They have been used in 65 studies (total N of patients = 8,522). They assess one or more aspects of social networks such as their size, structure, dimensionality and quality. Most instruments have various shortcomings, including questionable inter-rater and test-retest reliability.

**Conclusions:**

The assessment of social networks in patients with psychotic disorders is characterized by a variety of approaches which may reflect the complexity of the construct. Further research on social networks in patients with psychotic disorders would benefit from advanced and more precise instruments using comparable definitions of and timescales for social networks across studies.

## Introduction

Social network is a term used to describe the social ties linking individuals to other individuals through communication [1]. It is a social structure comprised of sets of interactions and defined by relationships between individuals.

Evidence suggests that social networks are linked to the onset of psychotic disorders and treatment outcomes of patients. Social networks were found to deteriorate prior to contact with services as a consequence of periods of untreated psychosis [2, 3]. In the examination of precursors to onset and recovery for mental illness, low levels of social support and poor social networks were suggested to increase the probability of onset of illness and decrease the probability of recovery. Better social networks have been associated with more favourable quality of life and fewer hospitalisations [4, 5, 6, 7]. Assessing social networks can help to identify protective factors against relapse [8] with potential implications for long-term treatment plans. An accurate assessment of social networks in patients with psychotic disorders is therefore important.

For assessing social networks, a technique called social network analysis was originally developed by anthropologists in order to provide qualitative descriptions of living systems in complex societies [9, 10, 11]. This technique was later applied to the problem of discovering and assessing the social support available to individuals in stressful situations, including but not limited to individuals with psychotic disorders. Later on, empirical studies and theoretical work concerning social networks and psychotic disorders became predominantly focused on qualitative aspects [12].

An assessment of social networks in patients with psychotic disorders may have to consider that social networks in these patients can be different from social networks in the general population. Their social networks are substantially smaller than those of people without mental illness. In most studies, social network is reported to consist of less than 10 members [13, 1, 14], and patients report having fewer people to turn into a crisis and fewer friends [14]. Social networks of patients with psychotic disorders also tend to be less stable, and–over time–can become even more restricted and less capable of providing the degree and type of support required for community integration [15, 16].

In an early review, Jackson and Edwards [17] identified eight instruments measuring social networks and social support in psychotic disorders in thirteen studies, out of which only four measured social networks specifically. The authors criticised a poor reliability and validity of existing instruments.

The review of Jackson and Edwards [17] did not use a systematic search method, and did not include any literature published after 1989. Also, their review was not focused specifically on instruments for the networks of patients with psychotic disorders. We therefore aimed to conduct a new review addressing the above shortcomings, i.e. using a systematic method of searching studies and collecting the relevant information, including papers published since 1989, and focusing on standardised assessment instruments for patients with psychotic disorders.

## Methods

We conducted a systematic review of studies assessing social networks in patients with psychotic disorders and identified the standardised instruments that were used to assess specifically social networks in these studies.

### Search Strategy

A protocol was developed using the Preferred Reporting Items for Systematic reviews and Meta-Analysis statement [18]. The electronic databases Medline, Embase, PsychInfo, Web of Knowledge, British Nursing Index (BNI) and Cumulated Index to Nursing and Allied Health Literature (CINAHL) were searched. Search terms were a combination of social contact assessment descriptors and psychotic disorder patient descriptors (*social contact* OR *social assessment* OR *social network*) AND (*psychosis* OR schiz* OR “*psychotic disorder*”). Grey literature databases were searched using the above search terms and thus papers were identified searching their title, abstract and full text with the aforementioned search terms.

Studies were also identified through citations from relevant literature reviews looking at social networks in people with psychotic disorders and a key journal hand-search of articles related to social networks was conducted with the reference lists of relevant articles. In order to make sure that all relevant papers were retrieved, a citation tracking element was used as part of the search strategy: citations of relevant papers (those describing instruments that have been included in the review) were searched using the Web of Science data base (www.webofknowledge.com).

### Eligibility Criteria

We included studies that used measures aiming to assess social networks of people with a diagnosis of psychotic disorder. Studies that used alternative diagnostic classifications or self-report diagnoses were translated into the appropriate International Classification of Diseases (10^th^ version) code (F20-F29) [19].

As many relevant studies had diagnostically mixed samples, we decided to include them if at least 50% of patients had been diagnosed with a psychotic disorder, a criterion used in other reviews [20]. Psychotic disorders comprised all diagnoses equivalent to the ICD-10 category ‘schizophrenia and related disorders’ (F2).

We included studies that used any type of standardised social networks measurement, with the exception of studies assessing exclusively interactions between the patient and family members. We included studies of all designs, of any publication year, and with samples of all ages, genders, and nationalities.

In case of doubts on the inclusion of papers the first authors of these publications were contacted.

### Study selection, data extraction and analysis

**Fig 1:**
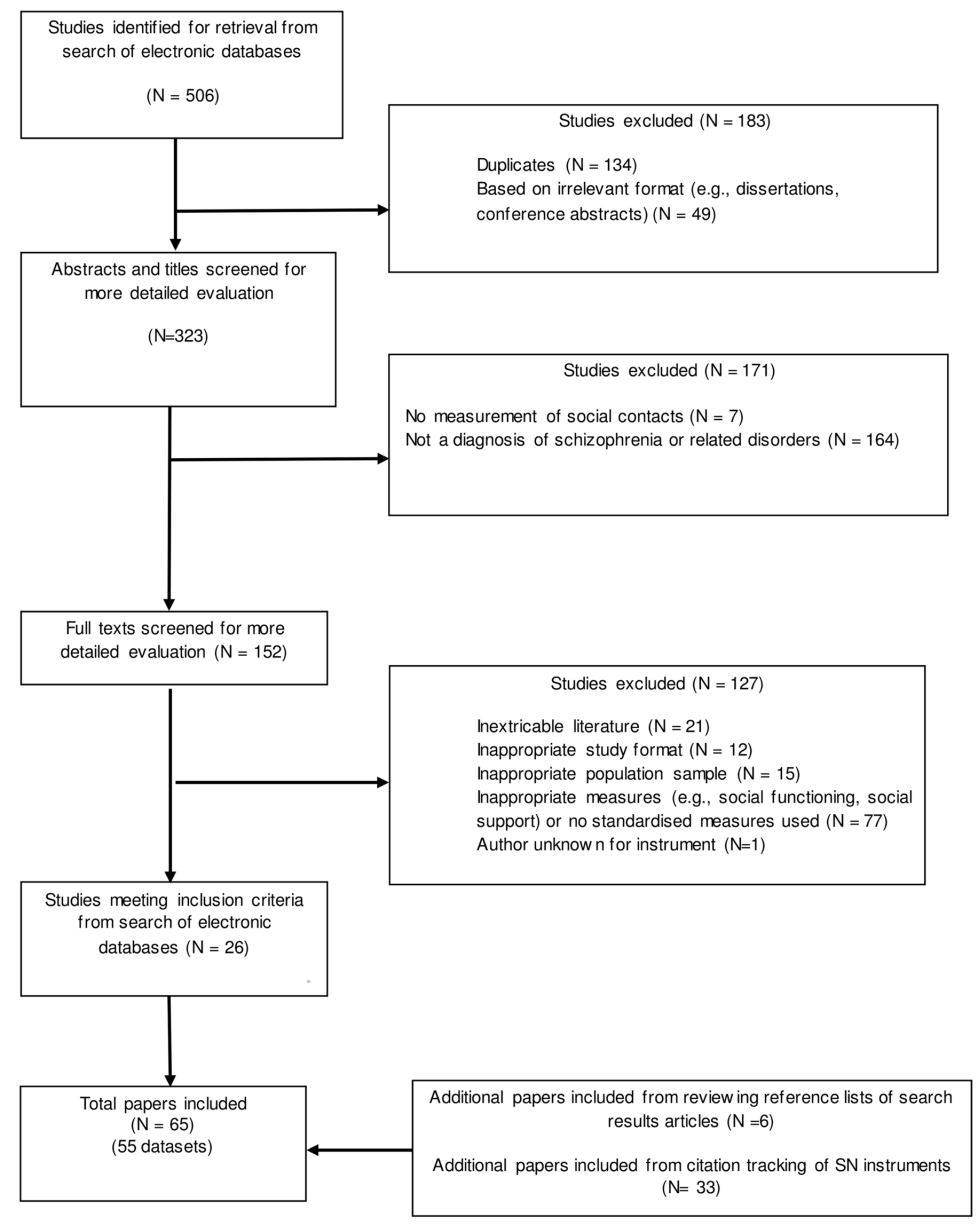
PRISMA Diagram.

All potential studies were exported into a reference citation manager and duplications removed. The author (JS) removed duplications and conducted the initial screening of titles and abstracts for inclusion. A random selection of 25% of the abstracts was then screened by the author (CG). If there was any ambiguity on the eligibility of the study, the full paper article was obtained and reviewed between the two authors (JS and CG). Inter-reviewer agreement was 90%, with disagreement on the inclusion of only one paper, which was brought to the third author (SP). Selected full-text articles were then obtained for the final screening. Final study selection was completed by two independent authors (JS and CG) with a third author (SP) helping to resolve disagreements. The details of the selection procedure are displayed in the PRISMA diagram (Fig 1).

Data extraction was completed by two reviewers (JS) and (CG) with a third reviewer adjudicating in the event of disagreement (SP). The data extraction tool was piloted to ensure proper documentation of the qualitative and quantitative components of the included studies.

Once finalized, data were extracted on study design, patient characteristics, assessment method, study findings, as well as extracting data specific for assessment (e.g. structure, time period, items, rater, results and emphasis). Main themes emerging from the papers and instruments were identified independently by JS and CG. When we could not obtain the original instruments, we attempted to assess their description in the included papers to reproduce the required information.

There was little inter-reviewer disagreement and any differences that were identified were resolved through discussion of the paper and the third reviewer (SP). JS and CG were in 100% agreement for all papers and instruments extracted and was not required to consult a third party (SP). For assessment instruments that could not be found, (CG) made direct contact requesting the necessary information to complete the synthesis. If the authors or relevant authority could not be contacted or did not respond, their papers and instruments were not included in this review (as a result, only one instrument–the Personal Network Interview [3] could not be included as the author for this instrument is unknown).

The papers were analysed descriptively.

## Results

### Selection of studies

A total of 509 records were retrieved. After the removal of duplicates and the application of inclusion and exclusion criteria on abstracts, 152 full-text papers were examined. After closer examination and conducting a final screen, as well as a citation tracking of relevant papers, 65 studies using standardised social networks instruments in schizophrenia were included in the review. Eight papers used data from one dataset, three papers included data from one dataset and another three included data from the same data set.

The selection of papers is shown in Fig 1. For characteristics of studies please see S1 Table.

Eight standardised social networks assessment tools were identified from these papers. The two most widely applied measures were the Interview Schedule for Social Interaction (ISSI) [21], used in 23 identified studies, and the Social Network Schedule (SNS) [8], applied in 21 studies.

### General characteristics of tools

The characteristics of the eight social networks instruments are summarised in Table 1. For four instruments we could not obtain the original schedules (ISSI, SNI, SSSNI, PPKI), however we judged that the description from included papers provided the required information.

**Table 1:** Measures of social networks in psychotic disorders.

Measure	Author	Country	What is measured?			Time period covered	Items	Result	Rater	Rating form*	Time to rate (min)	Studies	Properties	Emphasis
			Quantitative	Qualitative	Network members									
Arizona Social Support Schedule (ASSIS)	Barrera, 1981	USA	Available network size; utilised network size; network density, material aid, social participation, negative interactions	Support satisfaction, support need, advice, positive feedback,	Friends, family, others arising from intimate interaction, physical assistance	Previousmonth	24	Score	Researcher	Q	15–20	1	High test re-test reliability for both perceived and actual network size in general^1^ [22] and depressed sample^2^ [23]	Structure of network and satisfaction of support
Interview Schedule for Social Interaction (ISSI)	Henderson et al., 1980	Australia	Availability of attachment and social integration	Perceived adequacy of attachment and social integration	Friends, family, others	At present ×	50	Score	Trained Interviewer	Q, I	45	23	Adequate reliability and validity in a schizophrenia sample^3^ [16]	Availability of social relationships
Network Analysis Profile (NAP)	Sokolovsky and Cohen, 1981	USA	Size, density, degree, total network configurations(a modified version includes sustenance, reciprocity	Directionality, interactional content, intimacy	Family, friends, acquaintances, other	Previous month to hospitalisation	17 (modified version)	Score, mapping of network	Researcher	SSI	120	14	High inter-rater reliability in schizophrenia sample ^4^ [22]	Structure of network
Pattison Psychosocial Kinship Inventory (PPKI)	Pattison, 1981	USA	Size, multiplexity, ireciprocity **	Frequency of contacts	Family, relatives, friends, neighbours, co-workers, mental health professionals, acquaintance	At present ×	Not specified	Pictogram	Researcher	SSI	Not specified	2	Stability over 1 year and construct validity in a general population sample^5^ [24]	Structural, interactional and affective aspects of the social network.
Social Networks Interview (SNI)	Hammer 1981, Randolph 1982	USA	Size, percent kin, density, degree of linkages, reciprocity		Family, friends, neighbours, colleagues, others	Previous month to hospitalisation	Not specified	Network size, reciprocity rating, proportion and average number of linkages	Trained interviewer	SSI	90	1	None detailed	Structure of network
Social Network Schedule (SNS)	Dunn et al., 1990	UK	Size, frequency of communication (seen, telephone), percentage time spent socializing	Quality of relationship, intimacy, content of relationship, intensity of interaction	Relative, acquaintance, professional, neighbour, others	Previous month to hospitalisation	Not specified	Score or sociogram- a pictorial method of mapping the personal links between individuals	Researcher	SSI	15–20	19	High test re-test reliability at 10 days in a SMI sample ^6^ [8]	Network support
Social Relationship Scale (SRS)	McFarlane et al., 1981	Canada	Size, structure, reciprocity	Quality of network, content of relationship	Spouse, siblings, parents, other relatives, friends, physicians, colleagues, neighbours, others	Previous 12 months to hospitalisation	6	Score	Researcher	RS	90	3	Reliable over time, inadequate content validity, reasonable degree of criterion validity in a general population sample ^7^ [25]	Structure and quality of network
Social Support and Social Network Interview (SSSNI)	Lovell, Barrow and Hammer, 1984	USA	Size, density, frequency, degree, multiplexity	Quality of relationship, social support	People that provide support or service (relative, friends, acquaintances, professionals, other patients)	At present ×	6	Composit-ion: percentages density–ratio of linkages, multiplexity–average number of functions	Researcher	I	Not specified	2	Moderate test re-test reliability in a dual-diagnosis sample^8^ [26]	Social contact and support

* Rating form consists of interviews (I), semi-structured interview (SSI), structured interview (SI), questionnaire (Q), and rating (RS).

** Multiplexity refers to the number of different things done with a network member an instrumentality refers to how often each member provided emotional or practical support.

× It is suggested by the authors that “Time Period Covered” refers to the moment the measure was used, as not enough information was provided either in the description of the instrument, nor in any of the studies using it as to the exact timeline measured

^1^ Barrera et al., 1981

^2^ Barrera and Garrison-Jones, 1992

^3^ Bengtsson-Tops, 2004

^4^ Sokolovsky &Cohen, 1981

^5^ Jennings et al., 1988

^6^ Dunn et al. 10990

^7^ McFarlane et al., 1981

^8^ Goldberg et al. (2003)

The included assessments were published between 1972 and 1990. The total number of patients with psychotic disorders assessed using these tools was 8,522. The majority of instruments assess what the link is between the patient and their contacts across a certain time period. The most common type of assessment is a semi-structured interview although structured interviews and questionnaires were also used.

All assessment instruments are rated by an independent observer (with the exception of SRS and ASSIS that contain self-rated scales). The time taken to rate a patient’s social network ranges from ten minutes (e.g., SNS) to two and a half hours (e.g., NAP). The number of questions devoted to social contacts ranges from six (e.g. SRS) to 17 (e.g. NAP), with a total number of items ranging from eight to 50 items.

Most scales (seven out of eight) provide a quantifiable view of the network offering results in the form of either a score or percentage attributed to all social networks members (e.g.: how much of the network is made of family, friends, others) or a percentage of time spent socializing (the SNS). In terms of measuring satisfaction with different aspects of the network, three scales incorporated this aspect. The SRS and ASSIS require respondents to indicate their appraisal of each relationship using a Likert type rating scale, whilst the ISSI assesses satisfaction by asking patients whether the perceived amount of each relationship is sufficient.

The definition of a social contact is inconsistent across instruments and studies. For example, some instruments require patients to name all people they know (e.g. SNS) whilst others set a limit on the number of contacts respondents can name (e.g. SRS).

Instruments vary in the time period covered (i.e., from the past month to the past year) and contacts can be difficult to recall for lengthier time periods. Some measures require participants to name only those persons they had contact with during a specified time frame (the SNS, NAP, ASSIS and SNI specify the previous month), others do not provide a time frame (ISSI) whilst the SSSNI is unclear.

### Instruments

The most commonly used measures, the ISSI and the SNS, share a key conceptualisation of social networks as measuring both network structure and network quality variables.

The SNS has a strong focus on quantitative methodology which includes measures on different contact modalities (e.g., face to face, telephone) as well as the frequency of communication and the relationship to that contact, allowing for measuring total size of network as well as individual means for each component–size of network made of relatives, friends, confidants and so on).

A time budget, which provides a structure and helps patients recall their interactions is usually completed beforehand to elicit names of social contacts in the past month and six questions are asked for each of them (e.g., “How often do you see X?” and “Would you miss X if you never saw him/her again?”). The SNS was designed for inpatient populations and acknowledges the importance of transactions within wards (e.g. of lending and borrowing cigarettes) by differentiating between types of social interactions into conversational, non-verbal and salutatory. Reciprocity is thus assessed by acknowledging the behavioural significance assigned to each contact. Data gathered with this instrument can also be analyzed as a sociogram. Also, the SNS takes considerably less time to administer than the ISSI (15–20 minutes compared to 45 minutes) which may be an important factor when interviewing patients with psychotic disorders in lengthy research interviews.

The ISSI is split into four main scores: the availability of attachment (AVAT), the availability of social integration (AVSI), the perceived adequacy of attachment (ADAT), and the adequacy of social integration (ADSI). The first two categories (i.e. AVAT and AVSI) examine the quantitative aspects of social networks whereas the last two tap into the qualitative aspects and examine more closely the satisfaction with relationships, by asking participants whether the amount of each relationship available to them is appropriate or if they would like more or less of it. Whilst mean scores can be obtained for individual subsections, a total ISSI score can also be calculated and ranges from 0 to 30.

The Network Analysis Profile (NAP) [27] or modified versions, have been used in 15 studies. The NAP is a semi-structured interview that examines several aspects of social interaction including linkages between kin members, non-kin members, and formal members (e.g., agency). A modified version [28] further included structural dimensions (e.g., size, density, degree, clusters), interactional dimension (e.g., exchange, sustenance, directionality) and affective dimensions (e.g., positives including importance, friend, intimacy, reliability, satisfaction and negatives including critique, bossy, and intrusive) and the results can be interpreted either as a score or a pictogram. This instrument was particularly used in studies of older adults with psychotic disorders.

The other five instruments have been found to be used in only one to three studies.

The Social Relationship Scale (SRS) [25] provides a measure of patients’ appraisal of social support and the effectiveness of the support they receive on top of availability of support. The Adolescent Social Relationship Scale (ASRS) [26] was adapted from McFarlane et al.’s Social Relationship Scale [25] to measure the social networks of young people with and without psychosis and was used in only one study.

The Arizona Social Support Schedule (ASSIS) [22] assesses social network size, adequacy, and satisfaction alongside six social support functions (i.e, material aid, intimate interaction, feedback, guidance, physical assistance and social participation).

The Pattison Psychosocial Kinship Inventory (PPKI) [2] was one of the earliest instruments developed to identify the number of people, relationships, and interactions in social networks. Patients are asked to list those who were important to them at the time of interview.

The Social Support and Social Network Interview (SSSNI) [29] is based on four probe questions in order to identify the respondent’s network members (e.g.: “Who do you hang out for fun/relaxation?”, “Who would you go to for advice?”) followed by their functions as well as relationship with the member.

The Social Network Interview (SNI) [12] is a semi-structured interview covering information about interactions and starts off with questions about who they live with, contacts in their extended and nuclear family network, followed by specific questions to those who they feel close to.

### Reliability and Validity

Table 1 provides the available information about the psychometric properties of the instruments. Reliability and validity measures were not available for a number of instruments. Some were tested for stability (test-retest reliability, ISSI, ASSIS, PPKI, SRS; inter-rater reliability, SNS, NAP, SNI) and internal consistency (ISSI, PPKI, SRS), and some instruments were tested for construct and discriminant validity (SNS, ISSI). A few instruments were tested for both reliability and validity, using another measure of social contacts for validation purposes (SNS, ISSI).

## Discussion

### Main findings

We identified eight instruments that have been developed and used to assess social networks in patients with psychotic disorders. They were used in 65 studies comprising a total of more than 8,500 patients. Yet, they vary in the assessed variables, in their definitions of social networks and in the time frames they refer to when asking patients about their contacts. All of the instruments were published before 1992, and they commonly have some methodological shortcomings.

### Strengths and limitations

This review used a systematic method to search the literature for relevant studies and collate the findings. We found a substantial number of studies with data from over 8,500 patients, from different countries and from different types of studies. In order to minimise the possibility of missing relevant data, different researchers independently reviewed the data.

It is the first review of instruments assessing social networks in patients with psychotic disorders since 1992, and–to our knowledge–the first one at all using these systematic methods.

However, the review also has limitations. Firstly, we excluded one assessment tool for which the full text could not be found. Secondly, we included diagnostically mixed samples as long as at least 50% of patients were diagnosed with a psychotic disorder. And finally, we included only instruments that were explicitly specified to assess social networks. Thus, instruments that might assess relevant aspects of social networks, but use labels and terms other than social network were not considered. The rather restricted search terms may have contributed to the fact that we identified more relevant papers through citation tracking than in the first systematic search of databases (33 versus 26).

### Comparison with the literature

More than 20 years after the review of Jackson and Edwards [17], we still found that the concept of social network remains heterogeneous throughout the literature and that subsequently the definition of social network varies across instruments.

In the review of Jackson and Edward, four of the eight assessment instruments included in this review had not been considered (SNS, NAP, SNI and SSSNI), although only one of them has been published after 1989 (SNS), which is the final year considered in the review of Jackson and Edward. Of the 65 studies identified in this review however only six had been published before 1989. At the same time, Jackson and Edward had included instruments that we did not consider because of the narrower sole focus on social network assessments in this review.

Unlike Jackson and Edwards, we found that most scales incorporate both qualitative and quantitative social networks descriptors but because network quality is more methodologically problematic to assess, this infers a trade off in measurement accuracy. Due to differences between the quantitative versus qualitative components of social network assessed by the instruments, there is a high degree of variation of instruments included in this review and this makes comparison across studies difficult. Again, this reflects the lack of a unifying model of social networks in psychotic disorders.

### Choosing an instrument

The review did not identify a gold standard in the assessment of social networks in psychotic disorders. For selecting an instrument to assess social networks in patients with psychotic disorders in research and routine service evaluation, a number of aspects may need to be considered. Like in most selections of assessment instruments, there are general aspects. They include the purpose of the study, the role of the social network assessment in the study, the availability of data for relevant comparisons, the familiarity of the researchers with different instruments, practical issues such as the available time and training of the researchers and characteristics of the setting and the patient sample.

In case all these general aspects do not determine the choice, the ISSI and SNS as particularly useful instruments. They have been used in more studies than other instruments and therefore have more options for the direct comparison of data. They also have relatively well-established properties. Similar advantages may apply to the NAP which however takes considerably longer to administer. An interview of about two hours just for assessing one concept, i.e. social networks, may be seen as two long for most studies or routine evaluations. Yet, the ISSI and the SNS may be more appropriate for different types of studies.

In studies investigating interventions that focus on existing relationships and on strengthening interactions with core members of a social network, the ISSI may be preferred as it provides measures of the availability and perceived adequacy of social contacts.

For evaluating interventions that aim to increase the size and strength of social networks, the SNS may be more suitable as a measure of social network size and the presence of confidants and supportive relationships. Another advantage of the SNS is the name generator approach used at the beginning of the interview. This enables an alternative analysis of the data through a sociogram of the network. Such a visual representation allows to evaluate the centrality of the network and to tap into specific functional supportive roles of different contacts.

### Implications for future research

The current state of the art in assessing social networks of patients with psychotic disorders is limited due to the variety of understandings of the concepts and the absence of a consensus of the exact characteristics of a social network. This is reflected in the heterogeneity of existing instruments and the differences of approaches they use. Even when they refer to similar types of social contacts, they vary in the definition of contacts, the wording of questions, and the time frame to which the questions refer.

Some instruments use a patient’s subjective appraisal of the “importance” or “closeness” of a relationship as the sole criterion for including the given contact in their social network. However, such appraisal may be biased by memory effects and emotional emphases [30]. Additionally, this approach (i.e., patient’s appraisal of importance) may not correspond to the behavioural importance of that particular person as measured by exchanges of key goods, services or communication (e.g., “Who did you see yesterday?”, “What did you do together?”).

The different and sometimes non-specified periods of time that the questions refer to are linked with problems of recall. Usually the questions refer to at least one month. Recalling all contacts during the last month or more can be difficult. Much shorter time frames such as the last one or two days would facilitate more precise recall, but may provide less meaningful and representative data. Also, the different time frames hinder direct comparisons. For comparisons across studies, consistent time frames would be needed.

Further research aiming to improve the existing instruments or develop new ones may consider a few core requirements:

- the difficulties of defining social contacts and networks cannot be avoided and clarity is required even if the definitions are not perfect;
- there should be a clear distinction between objective behavioural measures (e.g. actual meetings) and subjective appraisals of a relationship (e.g. trust and closeness);
- the timeframes to which the questions relate should be specified; shorter time frames of a few days may be less representative for the life of the patients but provide information that is less influenced by memory bias; and
- future assessments will have to include interactions in social media and through the internet, which existing instruments (developed before 1992) do not consider at all.

## Conclusions

The number of included studies suggests an interest and a need to assess social networks of patients with psychotic disorders. Instruments for this purpose exist and have been used. Although the studies using them provide some valuable findings, the state of the art in assessing social networks in patients with psychotic disorders has not moved on since 1991 and further research is required to improve the feasibility and precision of instruments. Such research will have to accept the heterogeneity of the concepts of social networks and consider the specifics of social networks in patients with psychotic disorders. A consensus about core aspects such as wording and time frames would help to make findings comparable across studies, but will be difficult to achieve.

There may also be a need to have specified instruments for different purposes. Such purposes include assessing social networks in large scale epidemiological studies; as moderators, mediators or outcomes in clinical trials [31] or as criterion for planning and evaluating services in routine care. For any of these purposes, better instruments would be helpful and likely to stimulate wider assessments of social networks.

## Supporting Information

S1 Table Characteristics of Studies. (DOCX)

## References

[1] Cohen IC., Sokolovsky J. Schizophrenia and social networks: ex-patients in the inner city. Schizophrenia Bulletin, 1978, 4(4):546:560 734366 10.1093/schbul/4.4.546

[2] Jeppesen P; Petersen L; Thorup A; Abel MB; Ohlenschlaeger J; Christensen TO et al, The association between pre-morbid adjustment, duration of untreated psychosis and outcome in first-episode psychosis, Psychological Medicine, 2008, 38():1157–1166 10.1017/S0033291708003449 18447961

[3] Stein CH, Rappaport J, Seidman E, Assessing the social networks of people with psychiatric disability from multiple perspectives, 1995, Community Mental Health Journal, 31(4):351–367 7587155 10.1007/BF02207521

[4] Albert M, Becker T, Mccrone P, Thornicroft G. Social networks and mental health service utilisation—a literature review. International Journal of Social Psychiatry, 1998, 44(4):248–266 10459509 10.1177/002076409804400402

[5] Becker T, Leese M, Clarkson P, Taylor RE, Turner D, Kleckham J et al, Links between social networks and quality of life: an epidemiologically representative study of psychotic patients in South London, Social Psychiatry and Psychiatric Epidemiology, 1998, 33():299–304 10.1007/s001270050058 9689891

[6] Becker T, Leese M, Clarkson P, Taylor RE, Turner D, Kleckham J, Thornicroft G, Links between social networks and quality of life: an epidemiologically representative study of psychotic patients in South London, Social Psychiatry and Psychiatric Epidemiology, 1998, 33():299–304 10.1007/s001270050058 9689891

[7] Eklund M, Hansson L, Social network among people with persistent mental illness: associations with sociodemographic, clinical and health-related factors, International Journal of Social Psychiatry, 2007, 53(4): 293–305 17703645 10.1177/0020764006074540

[8] Dunn M, O'Driscoll C, Dayson D, Wills W, Leff J., The TAPS Project. 4: An observational study of the social life of long-stay patients, British Journal of Psychiatry, 1990, 157():842–848 2132311 10.1192/bjp.157.6.842

[9] Boissevain J. Network Analysis: A Reappraisal, Current Anthropology, 1979, 20 (3) pp 392–394

[10] Epstein A.L., 1961 The network and urban organisation Rhodes-Livingston Journal 29:29–62

[11] Mitchell J.C. 1969, ed Social networks in urban situations. Manchester: Manchester University Press pp 51–76.

[12] Randolph ET, Social networks and schizophrenia. In K.T Mueser & N. Tarrier (Eds), Handbook of Social Functioning in Schizophrenia (238–246).

[13] Bengtsson-Tops A; Hansson L, The validity of Antonovsky's Sense of Coherence measure in a sample of schizophrenic patients living in the community, Journal of Advanced Nursing, 2001, 33(4):432–438 11251730 10.1046/j.1365-2648.2001.01692.x

[14] MacDonald E.M, Hayes R., L, Baglioni A.J. Jr, The Quantity and quality of the social networks of young people with early psychosis compared with closely matched controls, Schizophrenia Research, 2000, 25:39 10.1016/s0920-9964(00)00024-4 11099882

[15] Becker T, Leese M, McCrone P, Clarkson P, Szmukler G, Thornicroft G, Impact of community mental health services on users' social networks. PRiSM Psychosis Study. 7. 1998, British Journal of Psychiatry 173():404–408 9926057 10.1192/bjp.173.5.404

[16] Bengtsson-Tops A, Mastery in patients with schizophrenia living in the community: relationship to sociodemographic and clinical characteristics, needs for care and support, and social network, 2004, Journal of Psychiatric and Mental Health Nursing, 11():298–304 15149377 10.1111/j.1365-2850.2003.00718.x

[17] Jackson HJ, Edwards J, Social networks and social support in schizophrenia: correlates and assessment, Schizophrenia–an overview and practical handbook, Chapman & Hall, London, 1992:275–292

[18] Moher D, Liberati A, Tetzlaff J, Altman DG, PRISMA Group, Preferred reporting items for systematic reviews and meta-analyses: the PRISMA statement. PLoS med 2009, 21(6): e1000097 10.1371/journal.pmed.1000097 Epub 2009 Jul 21. PMC2707599 19621072

[19] World Health Organisation, International Statistical Classification of Diseases and Related Health Problems, 10th Revision (ICD-10), 1992, Geneva: WHO

[20] Eassom E., Giacco D., Dirik A., Priebe S., Implementing family involvement in the treatment of patients with psychosis: a systematic review of facilitating and hindering factors, BMJ Open, 2014, 4:e006108 10.1136/bmjopen-2014-006108 25280809 PMC4187461

[21] Henderson S, Duncan-Jones P, Byrne DG, Scott R, Measuring social relationships The Interview Schedule for Social Interaction, Psychological Medicine, 1980, 10():723–734 7208730 10.1017/s003329170005501x

[22] Barrera M. Jr. Social support in the adjustment of pregnant adolescents In Gottlieb B.H. (Ed.), Social networks and social support. Beverly Hills: Sage, 1981, pp. 69–96.

[23] Barrera M Jr, Garrison-Jones C, Family and peer social support as specific correlates of adolescent depressive symptoms, 1992, Journal of Abnormal Child Psychology, 21(1): 1–16 10.1007/BF00927113 1548390

[24] Sokolovksy J, Cohen CI, Toward a Resolution of Methodological Dilemmas in Network Mapping, Schizophrenia Bulletin, 1981, 7(1):109–116 7233097 10.1093/schbul/7.1.109

[25] McFarlane AH, Neale KA, Norman GR, Roy RG, Streiner DL., Methodological Issues in Developing a Scale to Measure Social Support, 1981, 7(1):90–100 10.1093/schbul/7.1.90 7233115

[26] MacDonald E.M, Jackson H.J., Hayes R., L., Baglioni A.J, Madden C. Jr., Social skill as a determinant of social networks and perceived social support in schizophrenia, Schizoophrenia Research, 1996, 29(), 275–286 10.1016/s0920-9964(97)00096-0 9516669

[27] Pattison EM, Pattison ML, Analysis of a Schizophrenic Psychosocial Network, Schizophrenia Bulletin, 1981, 7(1):135–143 7233101 10.1093/schbul/7.1.135

[28] Cohen CI, Kochanowicz N, Schizophrenia and social network patterns: a survey of black inner-city outpatients, Community Mental Health Journal, 1989, 25(3):197–207 2805634 10.1007/BF00754437

[29] Lovell A. M., Barrow W., & Hammer M. (1984). Social Support and Social Network Interview. New York: New York State Psychiatric Institute.–

[30] O’Connor P, Brown GW., Supportive relationships: fact or fancy? Journal of Social and Personal Relationships, 1984, 1():159–175

[31] Bengtsson-Tops A, Hansson L. Quantitative and Qualitative Aspects of the Social Network in Schizophrenic Patients Living in the Community. Relationship To Sociodemographic Characteristics and Clinical Factors and Subjective quality of life, International Journal of Social Psychiatry, 2001, 47(3): 67–77 11589337 10.1177/002076400104700307

