# Supplementary material for: Assessing Social Networks in Patients with Psychotic Disorders: A Systematic Review of Instruments
Source: PLoS One. 2015 Dec 28;10(12):e0145250. doi: 10.1371/journal.pone.0145250 (PMC4692496; doi:10.1371/journal.pone.0145250)
Supplement: S1 Table — (DOCX) [file pone.0145250.s001.docx]

**Supplementary Table 1. Characteristics of Studies.**

| **Study** | **Country** | **Measure** | **Intervention** | **Primary Group** | **Group Size** | **Comparison Group** | **Group Size** | **Outcome** |
| --- | --- | --- | --- | --- | --- | --- | --- | --- |
| Abdallah et al. (2009) | USA | NAP | N/A | Schizophrenia (older adults) | 198 | General population | 113 | Compared to the comparison group, the schizophrenia sample had less community integration scores. |
| Albert et al. (2011) | Denmark | SNS | N/A | Schizophrenia and related disorders | 255 | - | - | Number of friends was predictive of recovery at 5 years follow up. |
| Angell and Test (1998) | USA | ISSI | N/A | Schizophrenia | 122 | - | - | Small mean network size for men and women, the latter had more contact with the opposite sex and for both genders social networks characteristics predicted satisfaction with social relationships. |
| Argentzell et al. (2014) | Sweden | ISSI | N/A | SMI*  (attending day centre ) | 40 | SMI*  (not attending day centre) | 67 | Day centre attendees had larger networks and a larger number of people they could ask to borrow things from. |
| Atkinson et al. (1996) | Scotland | SNS | Education | Schizophrenia | 73 | Schizophrenia | 57 | Intervention increased total number of contacts as well as number of confidants. |
| Bankole et al. (2006) | USA | NAP | N/A | Schizophrenia (older adults) | 198 | General population | 113 | The schizophrenia sample had less sustenance linkages than the general population sample and lower percentage of perceived reliable contacts. More reliable social contacts was associated with higher quality of life. |
| Bankole et al. (2008) | USA | NAP | N/A | Schizophrenia (older adults) | 198 | General population | 113 | Fewer social contacts was highly associated with remission in the schizophrenia sample. |
| Becker et al. (1997) | UK | SNS | N/A | SMI* | 143 | - | - | Increase in social network size decreased likelihood of hospitalization. Number of services used grew with social network size. |
| Becker et al. (1998a) | UK | SNS | N/A | Schizophrenia | 129 | - | - | Social networks are smaller in intensive service sector compared to standard service sector at 2 years. |
| Becker et al. (1998b) | UK | SNS | N/A | Schizophrenia | 195 | - | - | Quality of life positively related to number of social contacts. |
| Bengtsson-Tops (2001) | Sweden | ISSI | N/A | Schizophrenia out-patients | 120 | General population | 180 | Poorer network compared to normal sample in access, size and support. |
| Bengtsson-Tops (2004) | Sweden | ISSI | N/A | Schizophrenia | 94 | - | - | Changes in mastery was positively correlated to changes in access to social contacts. |
| Bengsstop- Tops & Hansson (2000) | Sweden | ISSI | N/A | Schizophrenia | 120 | - | - | An increased Sense of Coherence score was associated with a higher level of adequacy of social interaction. |
| Bengsstop- Tops & Hansson (2003) | Sweden | ISSI | New outpatient psychosis team | Schizophrenia (outpatients) – psychiatrist + supportive contact | 26 | Schizophrenia (outpatients) – psychiatrist contact | 24 | No difference in social network between the two groups. |
| Bjorkmann & Hansson (2000) | Sweden | ISSI | N/A | Schizophrenia and related disorders | 90 | - | - | More work time spend on indirect contacts on behalf of the client by case managers predicted an improved social network. |
| Bjorkmann & Hansson (2002) | Sweden | ISSI | N/A | Schizophrenia and related disorders | 67 | - | - | A decrease in symptoms and improvement in social networks predicted an improvement in subjective quality of life. |
| Bjorkmann & Hansson (2007) | Sweden | ISSI | N/A | Schizophrenia and related disorders | 60 | - | - | Scores on the ISSI were significantly higher at 6 years follow up for a cohort of patients with schizophrenia under a case management service. |
| Brunt and Hansson (2002) | Sweden | ISSI | N/A | SMI* inpatients | 23 | SMI* outpatients | 51 | No significant social networks differences between groups. Reported low scores on availability of social interaction in both groups as well and intermediate scores on the satisfaction of interaction. Emotional relationships were provided by immediate family and friends. There was also a positive correlation between ISSI score and the quality of life measure used in the study. |
| Catty et al. (2005a) | UK | SNS | N/A | SMI* day users of hospitals and centres | 169 | - | - | Longer duration of contact with services, more unmet needs, working and living in supported accommodation were associated with a larger network. Longer duration of contact was linked with having more confidantes. |
| Catty et al. (2005b) | UK | SNS | N/A | SMI* (attending day hospital) | 121 | SMI (attending day centre) | 160 | Day centre attendees had larger social networks and more intimate contacts than the comparison group. |
| Catty et al. (2012) | UK | SNS | N/A | SMI* | 53 | Anxiety, depression and personality disorders | 40 | Network size is not associated with therapeutic relationship ratings. |
| Clausen et al. (2014) | UK | SNS | N/A | First episode psychosis | 578 | - | - | People that had used cannabis had significantly less friends and family member they were in contact with. |
| Cohen et al. (2000) | USA | NAP | N/A | Schizophrenia (older adults) | 198 | General population | 113 | Number of confidants in the schizophrenia sample was not predictive of quality of life. |
| Cohen et al. (2008b) | USA | NAP | N/A | Schizophrenia (older adults) | 198 | General population | 113 | There was no statistically significant difference in number of sustenance links between older adults with schizophrenia who had suicide attempts in the past compared to those that hadn’t. |
| Cohen et al. (2011) | USA | NAP | N/A | Schizophrenia (older adults) | 198 | General population | 113 | Number of sustenance links did not mediate the effect of coping strategy on quality of life. |
| Cohen and Sokolovsky (1978) | USA | NAP | N/A | Schizophrenia | 22 | General population (matched) | 12 | Patients have smaller social networks than controls |
| Dayson et al. (1998) | UK | SNS | N/A | SMI* | 5 | SMI | 12 | Data collected at 5 year follow up with a sample of discharged patients into 2 homes (one significantly larger than the other) showed that there was more social cohesiveness in the smaller home. Reasons as to why residents failed to form relationships in larger homes remains unexplained. |
| Denoff and Pilkonis (1987) | USA | NAP-M | N/A | Schizophrenia | 103 | - | - | Premorbid social adjustment had little direct effect on network system development. |
| Diwan et al. (2007) | USA | NAP | N/A | Schizophrenia (adults) | 198 | General population | 113 | Proportion of confidants was associated with clinical depression in the schizophrenia group. |
| Eklund (2006) | Sweden | ISSI | N/A | SMI* | 60 | - | - | No difference between occupational groups (working, visiting activity centres and no regular activities) regarding qualitative or quantitative aspects of social networks. |
| Eklund and Hansson (2007) | Sweden | ISSI | N/A | SMI* outpatients | 103 | - | - | Higher levels of QOL, self-esteem, living in a house were related to higher ratings on the social network measuerments. |
| Eklund and Ostman (2010) | Sweden | ISSI | N/A | SMI* | 60 | - | - | There was a significant difference in aspects of social network between patients that scored lower on levels of satisfaction with sexual relations than those who scored higher with people from the latter group reporting larger social network and more satisfaction with daily activities/ |
| Erikson et al. (1998) | Canada | ISSI | N/A | SMI* | 48 | - | - | More supportive nonkin relations in the social network at the onset of schizophrenia predicted adaptive functioning 5 years after the first treatment. |
| Goddard et al. (2004) | UK | SNS | N/A | SMI* | 43 | SMI day patients | 78 | Social networks of discharged patients decreased. |
| Goldberg et al. (2003) | USA | SSSNI | N/A | SMI* outpatients | 219 | - | - | Network size (and not density) was related to quality of life, self-esteem and clinical symptomatology. |
| Hamilton et al. (1989) | USA | PPKI | N/A | Schizophrenia | 39 | - | - | Patients with more negative symptoms had smaller SNs. There were no correlations with positive symptoms. |
| Hansson et al. (2001) | Sweden | ISSI | N/A | Schizophrenia | 300 | - | - | The presence of unmet needs according to both patient and patient’s key worker was associated with a worse social network, |
| Hansson et al. (2002) | Nordic countries | ISSI | N/A | Schizophrenia outpatients | 418 | - | - | SNs was perceived as better by persons with an independent housing regardless if they were living alone or not, or with family or not. |
| Horan et al. (2006) | USA | SNI | N/A | Schizophrenia | 89 | - | - | Social network generally stable across 15 month follow-up. Smaller social networks related to poor current and premorbid social functioning. |
| Hultman et al. (1996) | Sweden | ISSI | N/A | Schizophrenia or schizophreniform disorder | 48 | - | - | Patients with a good social network at the time of admission were discharged sooner, had a faster recovery from clinical symptoms and these positive effects continued into remission. At relapse, patients who had good social integration had fewer reported symptoms but more observed negative symptoms. |
| Hultman et al. (1997) | Sweden | ISSI | N/A | Schizophrenia | 42 | - | - | People with low social integration had a higher relapse rate at 4 years than patients lacking of social provisions but wanting more. In a sub-sample of patients interviews for life events, the buffering effect of social factors, time between life event and relapse was significantly higher in patients with a high availability of attachment on the ISSI. |
| Ibrahim et al. (2010) | USA | NAP | N/A | Schizophrenia (adults) | 198 | General population | 113 | The number of intimate contacts was used as part of a way of measuring “successful aging” in a schizophrenia sample compared to controls. This number was significantly smaller in the schizophrenia group. |
| Jeppesen et al. (2008) | UK | SNS | N/A | First episode psychosis | 423 | - | - | Longer duration of untreated psychosis and poorer pre morbid social adaptation was associated with a small network at entry, 1 and 2 year follow up. |
| Joyce et al. (2000) | UK | SNS | N/A | Schizophrenia and related disorders | 69 | - | - | Total number of friends was predictive of the Experience of Caregiving Inventory (ECI). |
| Leff and Trieman (2000) | UK | SNS | N/A | SMI* | 523 | - | - | Size of patients’ network didn’t change at 5 years follow up. The number of confidants and friends however, increased in the first year, differing significantly from the baseline after 5 years in the community. |
| Lim et al. (2014) | USA | SRS | N/A | Psychosis | 25 | General population  New Religious Movement = 29 | 63 | Compared to the New Religious Movement group, people in the psychosis group had less crisis supports, unique supports, overalp supports and less helpful and reciprocal relationships. |
| Lindsted (2006) | Sweden | ISSI | N/A | Offenders with SMI* | 40 | - | - | Importance of daily activities was highly correlated with social participation measured by the ISSI. |
| Lipton et al. (1981) | USA | NAP-M | N/A | First-admission schizophrenia | 15 | Multiple admission schizophrenia | 15 | Networks of first-admission patients are larger and more inter-connected, have more multiplex and non-dependent links compared to multiple admission patients. |
| Macdonald et al. (1996) | Australia | SRS | N/A | First admission schizophrenia outpatients | 18 | Multiple admissions schizophrenia outpatients (history of 3 and more admissions) | 28 | Patients with more negative symptoms have smaller social support networks; patients who were more socially skilled have larger social networks ; younger people have larger networks than older people; no difference in perceiving support between all levels of social skilled patients/ young-old groups |
| Macdonald et al. (2000) | Australia | ASRS | N/A | Early psychosis | 26 | General population | 26 | Psychosis group had significantly smaller networks, fewer friends, fewer people to turn into a crisis and a higher likelihood of service providers as members. No difference in perceived social support, no of family members and no of participants with acquaintances. |
| Mattson et al. (2008) | Sweden | ISSI | N/A | First episode psychosis (recovered and unrecovered) | 71 | General population | 284 | Both recovered and non-recovered patients had smaller social networks compared to controls. Size, quality of network and perceived financial strain were predictive of the outcome. |
| Meeks and Murrell (1994) | USA | NAP | N/A | Schizophrenia and related disorders | 27 | General population | 19 | People with SMI have smaller networks which are less reciprocal and contained fewer family members |
| Middleboe (1997) | Denmark | SNS | N/A | SMI* | 37 | - | - | Number of reciprocal supportive contacts in the social network increased for a sample of patients with SMI part of a programme of small supportive group homes. |
| Mitchell (1989) | USA | ASSIS | N/A | SMI* outpatients | 150 | - | - | Social networks might be important in predicting mental health services utilization. |
| Nettelbladt et al. (1995) | Sweden | ISSI | N/A | Schizoaffective disorder | 18 | Diabetes patients =10. Normal controls =8 |  | Patients with schizoaffective disorder had less access to social relations and were less satisfied with their emotional relationships |
| Pattison and Pattison (1981) | USA | PPKI | N/A | Schizophrenia | 1 | - | - | Dynamics of a small network provide a pathogenic source of interpersonal relationships. |
| Pernice-Duca and Onaga (2009) | USA | SSSNI | N/A | SMI* | 221 | - | - | Network size decreased over time due to less professionals involved in care and recovery was positively correlated with qualitative aspects of network such as support, reciprocity. |
| Petersen et al. (2005) | Denmark | SNS | Integrated treatment | Schizophrenia and related disorders (integrated treatment) | 275 | Schizophrenia and related disorders (usual treatment) | 272 | There was no significant difference between the two groups regarding the median number of friends and family. |
| Sapra et al. (2006) | USA | NAP | N/A | Schizophrenia (older adults) | 198 | General population | 113 | The proportion of intimate contacts was not associated with any of the subscales of the Rating of Medication Influences Scale (ROMI) in the schizophrenia sample. |
| Soorgard et al. (2002) | Scandinavia (multicentre) | ISSI | N/A | Schizophrenia | 418 | - | - | Use of support contacts outside mental health professionals was associated with female sex, rural living and low GAF. |
| Sorgaard (2001) | Scandinavia (multicentre) | ISSI | N/A | Schizophrenia | 418 | - | - | Number of contacts related to high GAF, few BPRS negative and hostility symptoms, having contact with services and living in urban vs. rural areas. |
| Stein et al. (2013) | USA | SNS | N/A | SMI* | 60 | Parents of patients | 30 | Parents have more social networks than their children. Parents' reports of personal loss due to mental illness is related to their perceptions of social support. |
| Thornicroft & Breaky (1991) | UK | SNS | N/A | Schizophrenia | 97 | - | - | Patients in longer contact with COSTAR^[[1]](#footnote-1)^ programme had improved social function and in quality and quantity of SNs. |
| Thorup et al. (2006) | Denmark | SNS | Therapy, social skills training, family interven-tion | Schizophrenia (intervention) | 275 | Schizophrenia | 272 | Premorbid functioning, network size at entry and long duration of untreated psychosis is related to small SN size. Intervention was not able to address this problem. |
| Thorup et al. (2007) | Denmark | SNS | N/A | Schizophrenia | 578 | - | - | Men have poorer social networks than women. |

1. Community Support Treatment and Rehabilitation programme is a mobile treatment and case management service for SMI populations in Baltimore. Each patient is assigned a psychiatrist and nurse or social worker who work directly with them with a frequency of contact of 3 per week on an average

   SMI* = sample was >50% schizophrenia or related disorders
    [↑](#footnote-ref-1)
